# A New Nortriterpenoid, a Sesquiterpene and Hepatoprotective Lignans Isolated from the Fruit of *Schisandra chinensis*

**DOI:** 10.3390/molecules22111931

**Published:** 2017-11-10

**Authors:** Fenghua Li, Ting Zhang, Hua Sun, Haifeng Gu, Hongqing Wang, Xianming Su, Changkang Li, Baoming Li, Ruoyun Chen, Jie Kang

**Affiliations:** 1 State Key Laboratory of Bioactive Substance and Function of Natural Medicines, Institute of Materia Medica, Chinese Academy of Medical Sciences & Peking Union Medical College, No. 1 Xiannongtan Street, Beijing 100050, China; lifenghua@imm.ac.cn (F.L.); sunhua@imm.ac.cn (H.S.); wanghongqing@imm.ac.cn (H.W.); suxianming@imm.ac.cn (X.S.); lichangkang@imm.ac.cn (C.L.); libaoming@imm.ac.cn (B.L.); rych@imm.ac.cn (R.C.); 2 Institute of Medical Information & Library, Chinese Academy of Medical Sciences & Peking Union Medical College, No. 3 Yabao Street, Beijing 100020, China; brendatingting@126.com; 3 Beijing Institute for Drug Control, 25 Science Park Road, Changping District, Beijing 102206, China; ryan-ku@139.com

**Keywords:** *Schisandra chinensis*, nortriterpenoid, sesquiterpene, lignan, absolute configuration, hepatoprotective activity

## Abstract

A new nortriterpenoid, 19(*R*)-hydroxyl-wuweizidilactone H (**1**), and a sesquiterpene, (6*R*)-*β*-chamigrenic acid (**2**), together with one known nortriterpenoid, wuweizidilactone H (**3**), and three known hepatoprotective lignans, micrantherin A (**4**), gomisin M_2_ (**5**) and schizandrin (**6**) were isolated from the fruit of *Schisandra chinensis*. Their structures were elucidated by UV, IR, HRESIMS, NMR spectra and X-ray analysis. Among them, the absolute configuration of **2** was confirmed for the first time. In vitro assays, compounds **4**–**6** (10 μM) exhibited hepatoprotective activities (survival rate: 44%, 43% and 44%) against damage induced by *N*-acetyl-*p*-aminophenol (APAP) in human liver carcinoma (HepG2) cells.

## 1. Introduction

*Fructus schisandrae* (Wuweizi in Chinese), the fruit of *Schisandra chinensis* (Turcz.) Baill., is a traditional Chinese medicine (TCM), and has officially been used as an astringent tonic for more than two thousand years in China [1,2,3,4]. It is always recorded in the Chinese Pharmacopoeia. *S. chinensis* grows mainly in China, Japan, Korea and Eastern parts of Russia [1,5,6]. Outside China, the fruit of *S. chinensis* has had monographs in Japanese (2006), Korean (2002), Russian (1990) and American (1999) Pharmacopoeias [7,8]. A monograph on the fruit of *S. chinensis* has also been included in European Pharmacopoeia since 2008 [7,8]. The first official, internationally recognized monograph on this raw material has been available since 2007 in the international Pharmacopoeia edited by WHO [7,8]. Many types of compounds have been isolated from *S. chinensis*, including lignans, nortriterpenes, sesquiterpenes and phenolic acids. Some of them, especially dibenzocyclooctadiene lignans, have diverse liver healing properties [9,10]. Bifendate, an effective hepatoprotective drug used clinically for almost forty years, was derived from dibenzocyclooctadiene lignans of *S. chinensis* [11,12]. Although a lot of research on the fruit of *S. chinensis* was reported, it is still an interesting subject, and is a hot topic within medicinal chemistry and drug discovery community, and has been studied increasingly in recent years [13,14].

In the past decades, many efforts have been directed towards discovering the highly oxygenated nortriterpenes from the Schisandraceae family, which possess unprecedented carbon skeletons [15]. In our research, a new nortriterpenoid (18-norschiartane), 19(*R*)-hydroxyl-wuweizidilactone H (**1**), and a sesquiterpene, (6*R*)-*β*-chamigrenic acid (**2**) (the absolute configuration of which was confirmed for the first time), together with one known nortriterpenoid, wuweizidilactone H (**3**) [16], and three known hepatoprotective dibenzocyclooctadiene lignans, micrantherin A (**4**) [17], gomisin M_2_ (**5**) [18] and schizandrin (**6**) [19], were isolated from the fruit of *S. chinensis*. Herein, we report the details of the isolation, structure elucidation, and hepatoprotective activity of these compounds. 

## 2. Results and Discussion

### 2.1. Structural Analysis

**Figure 1:**
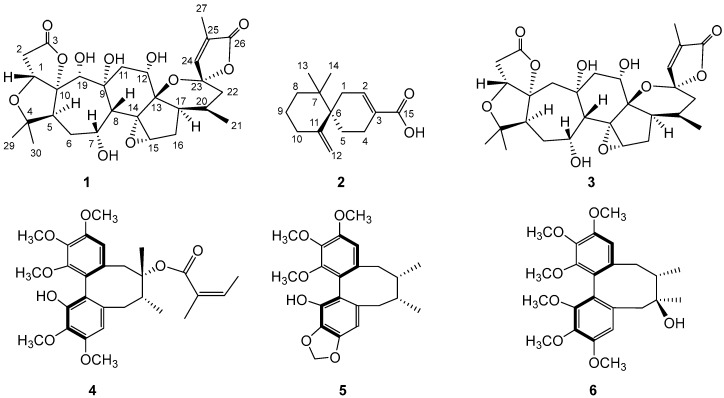
Structures of compounds **1**–**6**.

In this study, the CH_2_Cl_2_ fraction of the 75% EtOH extract of the fruit of *S. chinensis* yielded one new nortriterpenoid, 19(*R*)-hydroxyl-wuweizidilactone H (**1**), a sesquiterpene, (6*R*)-*β*-chamigrenic acid (**2**) (the absolute configuration of which was confirmed for the first time), one known nortriterpenoid, wuweizidilactone H (**3**), and three known hepatoprotective lignans, micrantherin A (**4**), gomisin M_2_ (**5**) and schizandrin (**6**) (Figure 1). The structures of these compounds were elucidated on the basis of spectroscopic or X-ray analysis.

**Figure 2:**
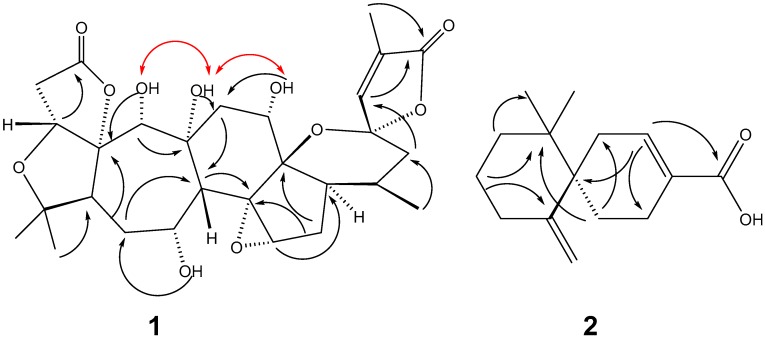
Key HMBC (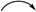) and NOE (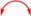) correlations of compounds **1** and **2**.

Compound **1**, obtained as white crystal (MeOH), m.p. > 200 °C, had a molecular formula of C_28_H_36_O_11_ derived from its HRESIMS spectrum at *m*/*z* 571.2156 [M + Na]^+^, and showed 11 degrees of unsaturation. The IR spectrum of **1** showed the presence of hydroxyl groups (3404 cm^−1^), a carbonyl group (1767 cm^−1^), and a double bond (1668 cm^−1^). The ^1^H- and ^13^C-NMR spectra displayed that **1** contained 28 carbons, including four methyl groups (one secondary and three tertiary carbons), five methylenes (five aliphatic carbons), ten methines (four oxygenated, one olefinic, and five aliphatic carbons), and nine quaternary carbons (two carbonyls, six oxygenated, and one olefinic carbon). These observations suggest that **1** has a highly oxygenated nortriterpenoid with a schisanartane skeleton that has eight rings, one double bond, and two carbonyl groups matching the observed degrees (11) of unsaturation. The analysis of NMR data of **1** and HRESIMS revealed that the structure of **1** was similar to that of wuweizidilactone H (**3**) [16], except for an extra hydroxyl group at C-19. The HMBC cross-peaks of H-19 (*δ*_H_ 4.22)/C-9 (*δ*_C_ 82.4) and 19-OH (*δ*_H_ 5.56)/C-10 (*δ*_C_ 101.5) determined this hydroxyl group’s position is at C-19. The positions of the other three hydroxyl groups were confirmed by the HMBC correlations of 7-OH (*δ*_H_ 4.61)/C-6 (*δ*_C_ 31.7), 9-OH (*δ*_H_ 4.62)/C-11 (*δ*_C_ 35.2) and 12-OH (*δ*_H_ 4.41)/C-11 (*δ*_C_ 35.2) (Figure 2). The strong NOE correlations of 19-OH/9-OH/12-OH determined the *β*-orientation of the H-19 (Figure 2). HRESIMS, UV, IR, 1D and 2D NMR spectra data see Supplementary data.

**Figure 3:**
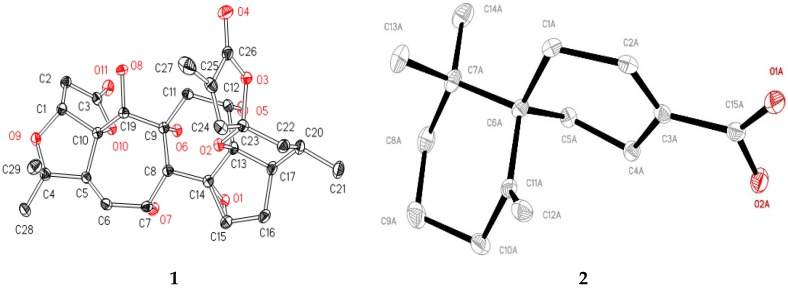
X-ray crystal structures of **1** and **2**.

The structure of **1** was completely determined by single crystal X-ray diffraction analysis (Figure 3). Accordingly, the structure of **1** was elucidated as 19(*R*)-hydroxyl-wuweizidilactone H (Figure 1).

Compound **2**, obtained as white crystals (MeOH), m.p. > 200 °C, showed a molecular ion peak in the HRESIMS spectrum at *m*/*z* 233.1542 [M − H]^−^, which was consistent with a molecular formula of C_15_H_22_O_2_ and indicative of five degrees of unsaturation. The data of ^1^H-, ^13^C-NMR and HMBC correlations (Figure 2) confirmed the planar structure of **2** is the same as *β*-chamigrenic acid [20]. The complete absolute configuration of C-6 in **2** was determined by single crystal X-ray diffraction analysis (Figure 3). Accordingly, the structure of **2** was elucidated as (6*R*)-*β*-chamigrenic acid (Figure 1). HRESIMS, UV, IR, 1D and 2D NMR spectra data see Supplementary data.

Though the structure of (6*S*)-*β*-chamigrenic acid had been shown in published papers, the absolute configuration of C-6 had not been confirmed by any data [20]. In addition, Scifinder recorded the structure of (6*R*)-*β*-chamigrenic acid (CAS 103089-10-7) from a Japanese patent [21], but actually, only the structure of (6*S*)-*β*-chamigrenic acid was shown in this patent [21], which was also not validated by any supporting data. 

The value of optical rotation of **2** is [*α*]${}_{D}^{25}$ −210.9 (*c* 0.1, CHCl_3_), which is similar to that of reported *β*-chamigrenic acid in the paper {[*α*]${}_{D}^{25}$ −134 (*c* 1.0, CHCl_3_)} [20] and in the patent {[*α*]${}_{D}^{25}$ −95.3 (*c* 0.43, CHCl_3_)} [21]. Only one chiral carbon was in the structure of *β*-chamigrenic acid. Thus, it was concluded that the complete reported absolute configuration of *β*-chamigrenic acid should be *R* (but not *S*), which is the same as that of **2**.

### 2.2. Hepatoprotective Activities of ***4**–**6***

To assess the biological activities of these three compounds, a human liver carcinoma cell (HepG2) injury model induced by *N*-acetyl-*p*-aminophenol (APAP) was adopted. Bicyclol, a hepatoprotective drug in clinic, was used as a positive control. As shown in Table 1, compounds **4**–**6** at a concentration of 10 μM showed moderate hepatoprotective activities.

About 40 lignans have been isolated from the fruit of *S. chinensis*, of which schizandrin (**6**) is one of the main lignans [8]. The good hepatoprotective effect of schizandrin (**6**) in vivo and in vitro has been studied before [22,23,24]. However, the hepatoprotective effect of micrantherin A (**4**) and gomisin M_2_ (**5**) has not been reported yet. From our experiment in vitro, micrantherin A (**4**) and gomisin M_2_ (**5**) showed a similar hepatoprotective effect as that of schizandrin (**6**), which suggested they also may be promising compounds for development of functional food materials beneficial to liver protection. The damaged HepG2 cell used in the hepatoprotective experiment model has been published in many papers [25,26,27], which suggests that this bioassay method is reliable. 

## 3. Materials and Methods

### 3.1. Plant Material

The cultivated fruit of *Schisandra chinensis* was collected from Ji’an County, Tonghua City, Jilin Province in September 2015, and was identified by Professor Lin Ma, Department of Natural Products Chemistry, Institute of Materia Medica (IMM), Chinese Academy of Medical Sciences and Peking Union Medical College (CAMS & PUMC), Beijing, China. A voucher specimen (ID-S-2864) was deposited in IMM, CAMS & PUMC.

### 3.2. Chemicals and Instruments 

Melting points were determined on a XT4-100B melting point apparatus (Jicheng Inc., Shanghai, China) and were uncorrected. Optical rotation was measured with a JascoP-2000 polarimeter (Tokyo, Japan). UV spectra were collected in MeOH on a JascoV-650 spectrophotometer (Tokyo, Japan). IR spectra were recorded on a Nicolet 5700 spectrometer (Madison, WI, USA) by the FT-IR transmission electron microscopy method. ^1^H- and ^13^C-NMR spectra were acquired using a Bruker-AvanceIII-400 (or 500) spectrometer (Bruker BioSpinGmBH, Rheinstetten, Germany) or an Agilent VNMRS600 (600 MHz) spectrometer (Palo Alto, CA, USA). HRESIMS were recorded on an Agilent 1200 SL series LC/6520 QTOF spectrometer (Boleblingen, Germany). Column chromatography (CC) purification was performed using silica gel (160–200 mesh, Qingdao Marine Chemical Factory, Qingdao, China), Sephadex LH-20 (GE Healthcare Bio-Sciences AB, Uppsala, Sweden), and C-18 (50 μm, YMC, Kyoto, Japan). The human hepatic carcinoma cell (HepG2) purchased from Shanghai Gefan Industrial Co., Ltd. (Shanghai, China). 

### 3.3. Extraction and Isolation

Air dried, powdered fruits of *S. chinensis* (28.4 kg) were extracted with EtOH–H_2_O (75:25, *v*/*v*, ×3) under reflux conditions for 1.5 h and concentrated under reduced pressure. The residue (3.1 kg) was suspended in H_2_O and successively partitioned with petroleum ether, CH_2_Cl_2_, EtOAc, and *n*-BuOH. The CH_2_Cl_2_ extract (800 g) was chromatographed over silica gel (160–200 mesh) and eluted with a gradient system of petroleum ether/acetone (100:0 → 30:70, *v*/*v*). The 15 fractions were collected. After removal of solvents, fraction 3 (60 g) was applied to an ODS column, eluted with a gradient elution of MeOH/H_2_O (40:60 → 100:0, *v*/*v*), then was fractionated by chromatography on Sephadex LH-20 column (MeOH/H_2_O, 8:2, *v*/*v*) and further purified by semi-preparative HPLC to afford compounds **2** (50 mg, MeOH/H_2_O, 6:4, *v*/*v*) and **5** (100 mg, MeOH/H_2_O, 7:3, *v*/*v*). Fraction 8 (10 g) was subjected to a Sephadex LH-20 column (MeOH/H_2_O, 7:3, *v*/*v*) and then purified by semi-preparative HPLC, using an isocratic elution (MeOH/H_2_O, 6:4, *v*/*v*) to obtain **4** (13 mg) and **6** (15 mg). Fraction 14 (1.1 g) was purified by semi-preparative HPLC (MeOH/H_2_O, 5:5, *v*/*v*) to yield **1** (100 mg) and **3** (500 mg).

### 3.4. Characterization of Compounds ***1**–**2***

#### 3.4.1. 19(*R*)-Hydroxyl-Wuweizidilactone H (**1**)

White crystal (MeOH), m.p. > 200 °C; [*α*]${}_{D}^{20}$ +34.9 (*c* 0.1, MeOH); UV (MeOH) *λ*_max_ (log *ε*) 206 (1.26) nm; IR *ν*_max_ 3404, 1767, 1668, 1445 cm^−1^. For ^1^H-NMR (DMSO-*d*_6_, 600 MHz) and ^13^C-NMR (DMSO-*d*_6_, 150 MHz) spectroscopic data, see Table 2. HRESIMS (positive ion) *m*/*z* 571.2156 [M + Na]^+^ (calcd. for C_28_H_36_NaO_11_, 571.2150).

Crystallographic Data for **1**: Monoclinic, *a* = 13.7591(2) Å, *b* = 15.1229(4) Å, *c* = 14.0956(5) Å, *α* = 90.00°, *β* = 101.459(2)°, *γ* = 90.00°, V = 2874.51(14) Å^3^, T = 101 K, space group P2_1_/*n*, Z = 4, 10,924 reflections collected, 5456 independent reflections; final R indexes [I > 2*σ* (I)] R1 = 0.0414, wR2 = 0.1073; final R indexes (all data) R1 = 0.0479, wR2 = 0.1133. The goodness of fit on F2 was 1.023. The crystallographic data for 19(*R*)-hydroxy-wuweizidilactone H (**1**) were deposited at the Cambridge Crystallographic Data Centre (deposition No. CCDC 1576015) and can be obtained free of charge via www.ccdc.cam.ac.uk/deposit (or from the CCDC, 12 Union Road, Cambridge cb21EZ, UK; fax: +44-1223-336033; deposit@ccdc.cam.ac.uk).

#### 3.4.2. (6*R*)-*β*-Chamigrenic Acid (**2**)

White crystal (MeOH), m.p. > 200 °C; [*α*]${}_{D}^{20}$ −210.9 (*c* 0.1, CHCl_3_); UV (MeOH) *λ*_max_ (log *ε*) 242 (1.26) nm; IR *ν*_max_ 2928, 1680, 1425, 1274 cm^−1^. For ^1^H-NMR (CD_3_OD, 600 MHz) and ^13^C-NMR (CD_3_OD, 150 MHz) spectroscopic data, see Table 2. HRESIMS (negative ion) *m*/*z* 233.1542 [M − H]^−^ (calcd. for C_15_H_21_O_2_, 233.1547).

Crystallographic Data for **2**: Monoclinic, *a* = 13.7591(2) Å, *b* = 15.1229(4) Å, *c* = 14.0956(5) Å, *α* = 90.00°, *β* = 101.459(2)°, *γ* = 90.00°, V = 2874.51(14) Å^3^, T = 101 K, space group P2_1_/n, Z = 4, 10,924 reflections collected, 5456 independent reflections; final R indexes [I > 2*σ* (I)] R1 = 0.0414, wR2 = 0.1073; final R indexes (all data) R1 = 0.0479, wR2 = 0.1133. The goodness of fit on F2 was 1.023. The crystallographic data for (6*R*)-*β*-chamigrenic acid (**2**) were deposited at the Cambridge Crystallographic Data Centre (deposition No. CCDC 1576013) and can be obtained free of charge via www.ccdc.cam.ac.uk/deposit (or from the CCDC, 12 Union Road, Cambridge cb21EZ, UK; fax: +44-1223-336033; deposit@ccdc.cam.ac.uk).

### 3.5. Hepatoprotective Effects of Compounds on Damaged HepG2 Cells Induced by APAP

The hepatoprotective effects of compounds **4**–**6** against damage induced by APAP in human liver carcinoma (HepG2) cells were determined by the MTT colorimetric assay as previously described [26,28]. Each cell suspension of 2 × 10^4^ cells in 200 μL of RPMI 1640 containing fetal calf serum (10%), penicillin (100 U/mL), and streptomycin (100 μg/mL) was placed in a 96-well microplate and pre-cultured for 24 h at 37 °C under a 5% CO_2_ atmosphere. Fresh medium (100 μL) containing bicyclol and test samples were added, and the cells were cultured for 1 h. Then, the cultured cells were exposed to 25 mM DL-galactosamine for 24 h. Then, 100 μL of 0.5 mg/mL MTT was added to each well after the withdrawal of the culture medium and incubated for an additional 4 h. The resulting formazan was dissolved in 150 μL of DMSO after aspiration of the culture medium. The optical density (OD) of the formazan solution was measured on a microplate reader at 492 nm.

## 4. Conclusions

In this study, three dibenzocyclooctadiene lignans, including micrantherin A (**4**), gomisin M_2_ (**5**) and schizandrin (**6**) all exhibited hepatoprotective activities (survival rate: 44%, 43% and 44%) against damage induced by APAP in human liver carcinoma (HepG2) cells. However, nortriterpenoids 19(*R*)-hydroxyl-wuweizidilactone H (**1**) and wuweizidilactone H (**3**) did not show hepatoprotective activities in the experiment.

The dibenzocyclooctadiene lignans are the main constituents in *S. chinensis*, and have attracted a lot of attention and been studied extensively since the 1970s [29,30,31]. Varied bioactivities of dibenzocyclooctadiene lignans, such as hepatic protection, anti-hepatitis B virus, anti-inflammation and cytotoxic activity have been reported previously [3,5,32,33]. However, no more than 20 nortriterpenoids have been found in *S. chinensis* since 2007, and only cytotoxic activity was reported [28]. Thus, the more nortriterpenoids and their related bioactivities need to be further studied extensively.

## Figures and Tables

**Table 1:** Hepatoprotective effects of compounds **4**–**6** (10 μM) on the survival rate of HepG2 cell injured by APAP.

Compounds	OD Value	Survival Rate (%)
Blank control	1.957 ± 0.116	100.0
Model (APAP 8 mM)	0.759 ± 0.012 ***	38.7
Bicyclol (positive control)	1.061 ± 0.053 ^##^	50.6
**4**	1.061 ± 0.053 ^##^	44.5
**5**	1.061 ± 0.053 ^##^	43.5
**6**	1.061 ± 0.053 ^##^	44.6

*** *p <* 0.001 vs. blank; ^##^ *p <* 0.01 vs. model.

**Table 2:** ^1^H- (600 MHz) and ^13^C-NMR (150 MHz) data of **1** and **2**.

Position	Compound 1 ^a^		Compound 2 ^b^	
	^13^C	^1^H- (*J* Hz)	^13^C	^1^H- (*J* Hz)
1	80.9	4.34 m	31.5	2.30 m (2 H)
2	37.0	2.18 d (17.6) 2.82 dd (17.6, 6.0)	141.5	6.96 s
3	175.9	---	131.5	---
4	83.1	---	38.5	1.86 m (2 H)
5	50.9	2.42 dd (13.6, 1.2)	27.3	1.44 m, 2.14 m
6	31.7	1.86 m, 1.55 m	46.8	---
7	67.4	3.99 m	38.7	---
7-OH	---	4.61 m	---	---
8	42.2	2.26 m	25.4	1.52 m, 1.61 m
9	82.4	---	23.8	1.71 m, 2.30 m
9-OH	---	4.62 m	---	---
10	101.5	---	33.7	2.11 m, 2.30 m
11	35.2	2.21 m, 1.55 m	150.7	---
12	75.4	3.65 m	111.9	4.89 s, 4.43 s
12-OH	---	4.41 m	---	---
13	83.2	---	25.9	0.88 s (3 H)
14	69.3	---	21.4	0.92 s (3 H)
15	53.2	3.75 brs	170.5	---
16	24.2	1.86 m, 1.65 m		
17	33.1	2.42 m		
19	67.1	4.22 m		
19-OH	---	5.56 m		
20	21.6	2.10 m		
21	18.7	0.91 d (6.4, 3 H)		
22	33.7	1.40 m, 1.41 dd (14, 3.6)		
23	105.4	---		
24	148.9	7.11 s		
25	130.3	---		
26	171.2	---		
27	10.2	1.80 s (3H)		
29	22.1	1.04 s (3H)		
30	28.1	1.19 s (3H)		

Measured ^a^ in DMSO-*d*_6_, ^b^ in CD_3_OD.

## Supplementary Materials

Supplementary data associated with this article, including HRESIMS, UV, IR, 1D and 2D NMR spectra data (**1** and **2**) can be found in the online version.
