# Supplementary material for: A New Nortriterpenoid, a Sesquiterpene and Hepatoprotective Lignans Isolated from the Fruit of Schisandra chinensis
Source: Molecules. 2017 Nov 10;22(11):1931. doi: 10.3390/molecules22111931 (PMC6150265; doi:10.3390/molecules22111931)
Supplement: Supplementary file 1 [file molecules-22-01931-s001.pdf]

molecules

Supplementary Information

MS Formula Results: + Scan (6.330 min) Sub (2017030601.d)

m/z	Ion	Formula	Abundance
571.2156	(M+Na)+	C28 H36 Na O11	16594.6

Best	Formula (M)	Ion Formula	Score	Cross Sco	Mass	Calc Mass	Calc m/z	Diff (ppm)	Abs Diff (ppm)	Mass Match	Abund Match	Spacing Match	DBE
✓	C28 H36 O11	C28 H36 Na O11	98.98		548.2264	548.2258	571.215	-1.11	1.11	99.96	98.44	97.64	11
	C29 H32 N4 O7	C29 H32 N4 Na O7	98.56		548.2264	548.2271	571.2163	1.31	1.31	99.94	97.1	97.52	16
	C25 H40 O11 S	C25 H40 Na O11 S	98.09		548.2264	548.2291	571.2184	5.02	5.02	99.19	99.12	94.65	6
	C20 H40 N2 O13 S	C20 H40 N2 Na O13 S	97.6		548.2264	548.2251	571.2143	-2.33	2.33	99.83	96.76	94.15	2
	C32 H36 O6 S	C32 H36 Na O6 S	97.5		548.2264	548.2233	571.2125	-5.69	5.69	98.96	96.97	95.23	15
	C33 H32 N4 O2 S	C33 H32 N4 Na O2 S	97.29		548.2264	548.2246	571.2138	-3.26	3.26	99.66	95.14	95.12	20
	C29 H40 O6 S2	C29 H40 Na O6 S2	97.19		548.2264	548.2266	571.2159	0.45	0.45	99.99	95.61	93.49	10
	C30 H36 N4 O2 S2	C30 H36 N4 Na O2 S2	96.88		548.2264	548.228	571.2172	2.88	2.88	99.73	95.04	93.37	15
	C38 H32 N2 S	C38 H32 N2 Na S	95.95		548.2264	548.2286	571.2178	4.08	4.08	99.46	90.38	95.62	24
	C41 H28 N2	C41 H28 N2 Na	95.28		548.2264	548.2252	571.2145	-2.06	2.06	99.86	85.43	97.94	29

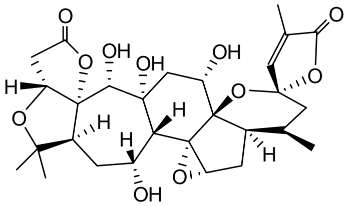

Figure S1. HRESIMS spectrum of 19(*R*)-hydroxyl-wuweizidilactone H (1)

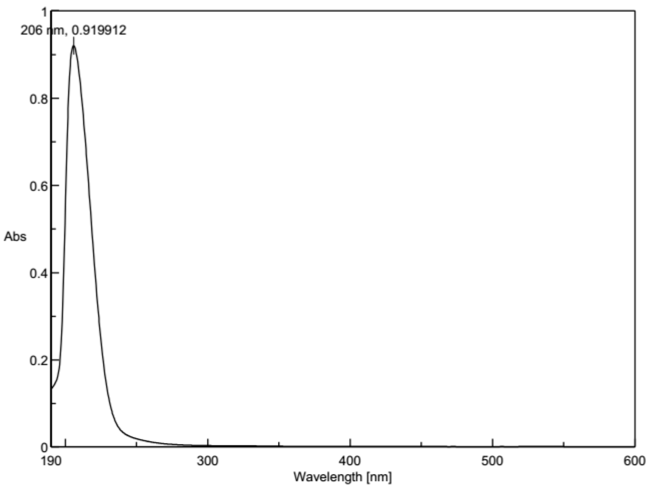

Memory-4

[Comment]
Sample Name sc-4-123-2
Comment 0.05
User
Division UV
Company 324

[Measurement Information]
Instrument Name V-650
Model Name V-650
Serial No. A034461150

Accessory PSC-718
Accessory S/N A001761114
Position 1
Cell Length 10 mm
Temperature 19.98 C
Control Sonsor Holder
Monitor Sensor Holder
Start Mode Start immediately

Photometric Mode Abs
Measurement range 600 - 190 nm
Data pitch 0.2 nm
Band width(UV/Vis) 2.0 nm
Response Medium
Scanning speed 200 nm/min
Source Change 340 nm
Light Source D2/WI
Filter Exchange Step
Correction Baseline

[Data Information]
Creation Date 2017-3-7 17:18

Data array type Linear data array
Horizontal Wavelength [nm]
Vertical Abs
Start 600 nm
End 190 nm
Data pitch 0.2 nm
Data points 2051

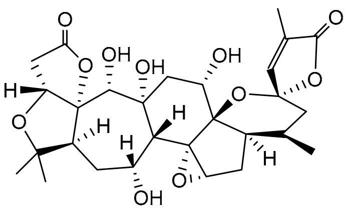

Figure S2. UV spectrum of 19(*R*)-hydroxyl-wuweizidilactone H (1)

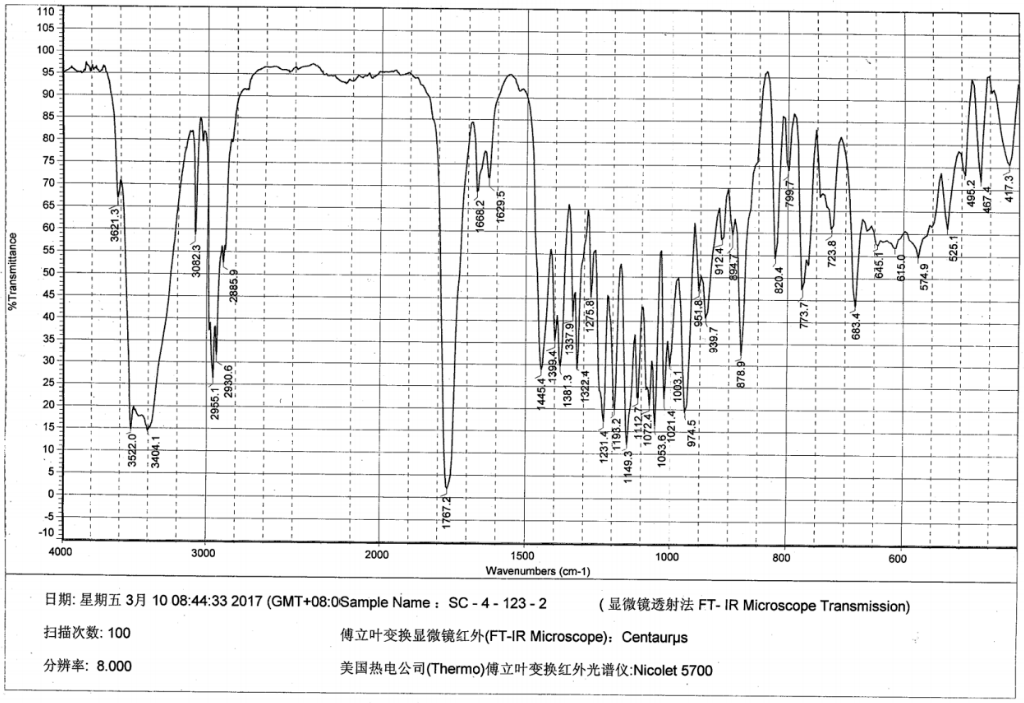

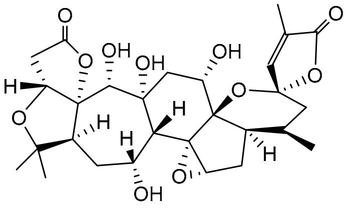

Figure S3. IR spectrum of 19(*R*)-hydroxyl-wuweizidilactone H (1)

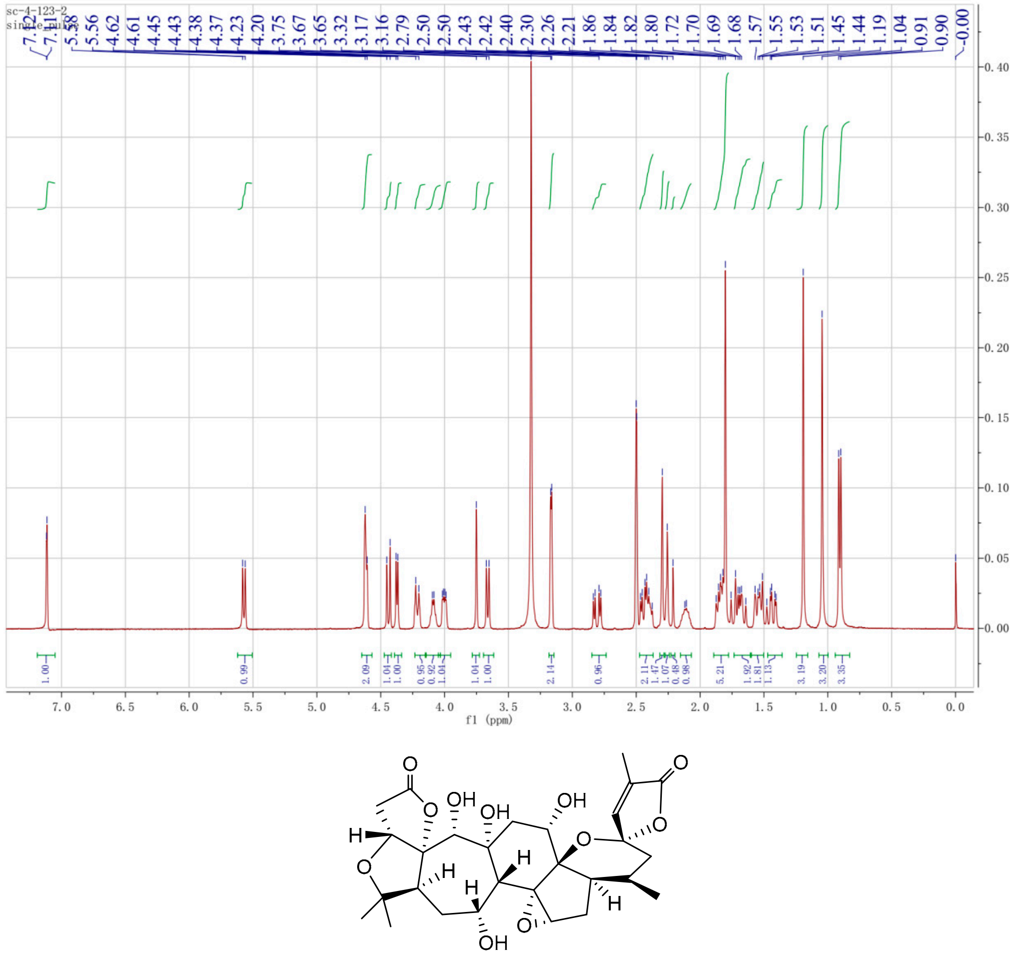

Figure S4. ^{1}H NMR spectrum of 19(*R*)-hydroxyl-wuweizidilactone H (1; 600 MHz, DMSO-d_6)

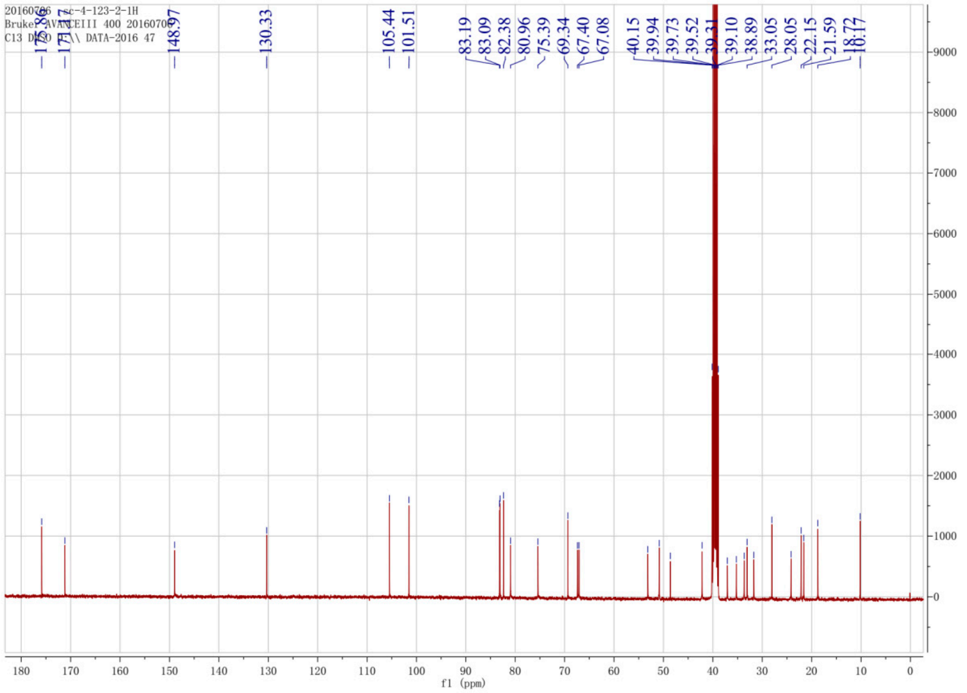

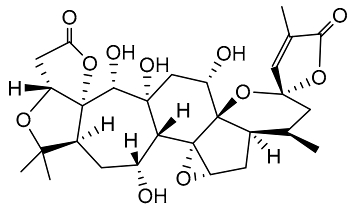

Figure S5. ^{13}C NMR spectrum of 19(*R*)-hydroxyl-wuweizidilactone H (1; 150 MHz, DMSO-d_6)

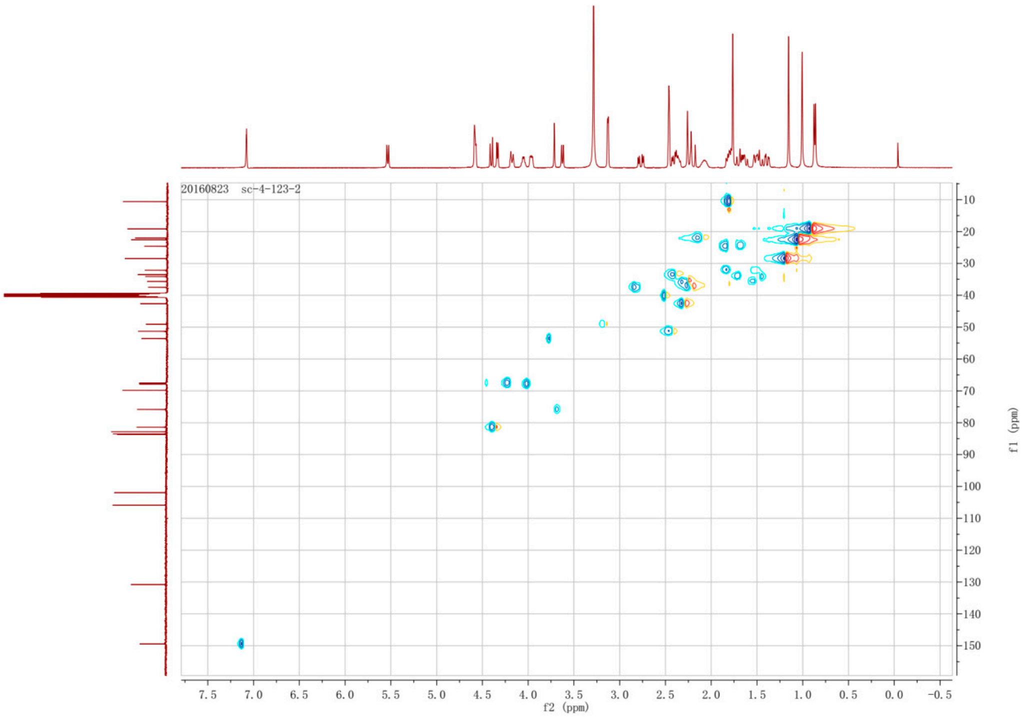

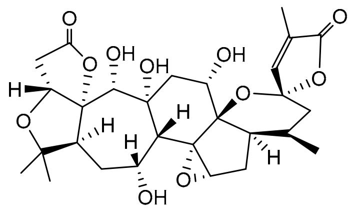

Figure S6. HSQC spectrum of 19(*R*)-hydroxyl-wuweizidilactone H (1; DMSO-d_6)

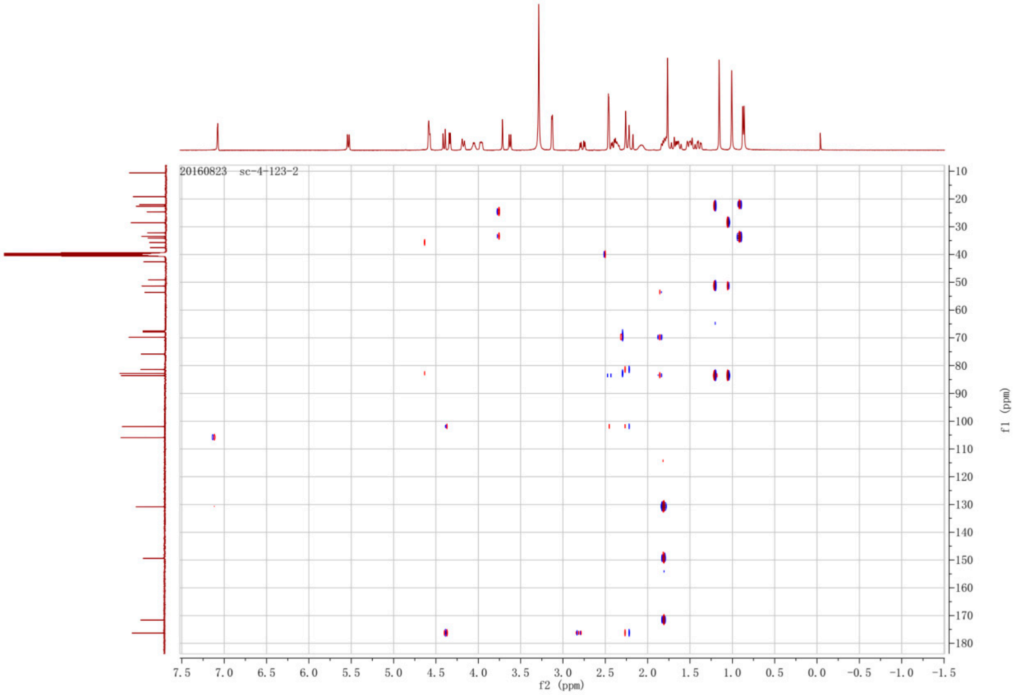

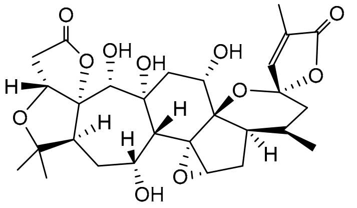

Figure S7. HMBC spectrum of 19(*R*)-hydroxyl-wuweizidilactone H (1; DMSO-*d*6)

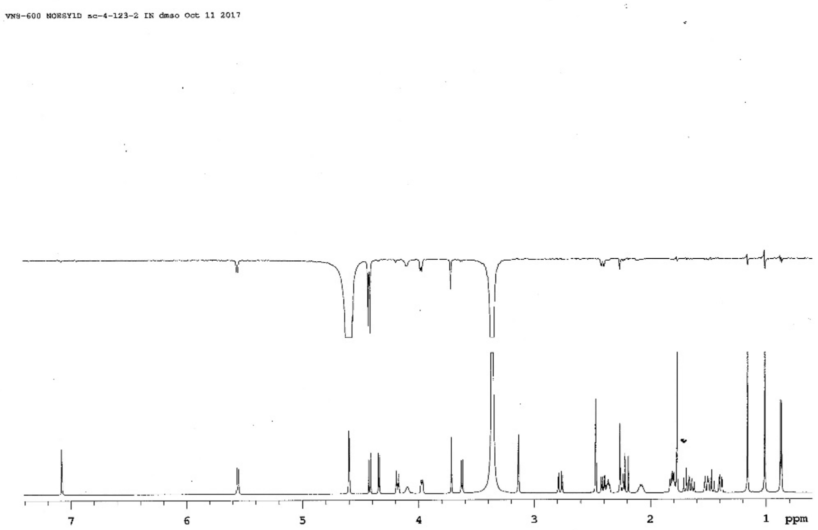

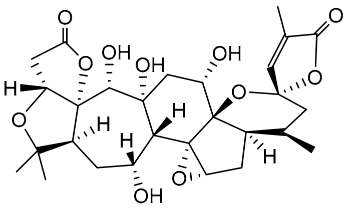

Figure S8. 1D NOSEY spectrum of 19(*R*)-hydroxyl-wuweizidilactone H (1; DMSO-d_6)

MS Formula Results: - Scan (8.364 min) Sub (2017031602.d)

m/z	Ion	Formula	Abundance
233.1542	(M-H)-	C15 H21 O2	76161.7

Best	Formula (M)	Ion Formula	Score	Cross Sco	Mass	Calc Mass	Calc m/z	Diff (ppm)	Abs Diff (ppm)	Mass Match	Abund Match	Spacing Match	DBE
☑	C15 H22 O2	C15 H21 O2	99.77		234.1615	234.162	233.1547	2.16	2.16	99.86	99.49	99.93	5
☐	C10 H22 N2 O4	C10 H21 N2 O4	96.64		234.1615	234.158	233.1507	-15.02	15.02	93.58	98.95	100	1
☐	C12 H26 O2 S	C12 H25 O2 S	95.4		234.1615	234.1654	233.1581	16.55	16.55	92.26	97.09	99.64	0
☐	C9 H22 N4 O3	C9 H21 N4 O3	86.58		234.1615	234.1692	233.1619	32.94	32.94	72.69	98.61	99.93	1
☐	C9 H22 N4 O S	C9 H21 N4 O S	78.98		234.1615	234.1514	233.1442	-42.9	42.9	58.22	96.54	99.42	1
☐	C14 H22 N2 O	C14 H21 N2 O	75.02		234.1615	234.1732	233.1659	50.13	50.13	47.78	99.63	99.99	5
☐	C12 H18 N4 O	C12 H17 N4 O	70.5		234.1615	234.1481	233.1408	-57.29	57.29	38.11	99.94	99.98	6

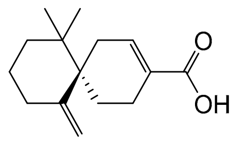

Figure S9. HRESIMS spectrum of (6*R*)-*β*-chamigrenic acid (2)

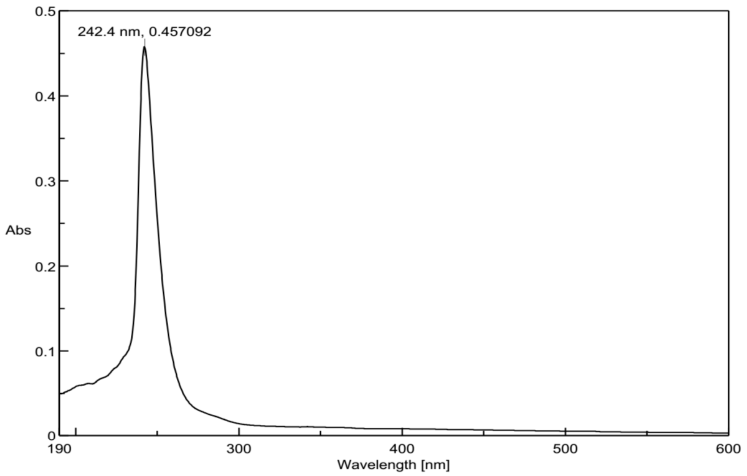

[Comment]
Sample Name sc-4-27-30
Comment 0.025
User
Division UV
Company 324
[Measurement Information]
Instrument Name V-650
Model Name V-650
Serial No. A034461150

Accessory PSC-718
Accessory S/N A001761114
Position 1
Cell Length 10 mm
Temperature 19.97 C
Control Sensor Holder
Monitor Sensor Holder
Start Mode Start immediately

Photometric Mode Abs
Measurement range 600 - 190 nm
Data pitch 0.2 nm
Band width(UV/Vis) 2.0 nm
Response Medium
Scanning speed 200 nm/min
Source Change 340 nm
Light Source D2/WI
Filter Exchange Step
Correction Baseline

[Data Information]
Creation Date 2017-7-4 16:36

Data array type Linear data array
Horizontal Wavelength [nm]
Vertical Abs
Start 600 nm
End 190 nm
Data pitch 0.2 nm
Data points 2051

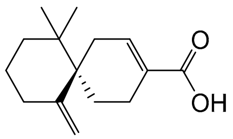

Figure S10. UV spectrum of (6*R*)-*β*-chamigrenic acid (2)

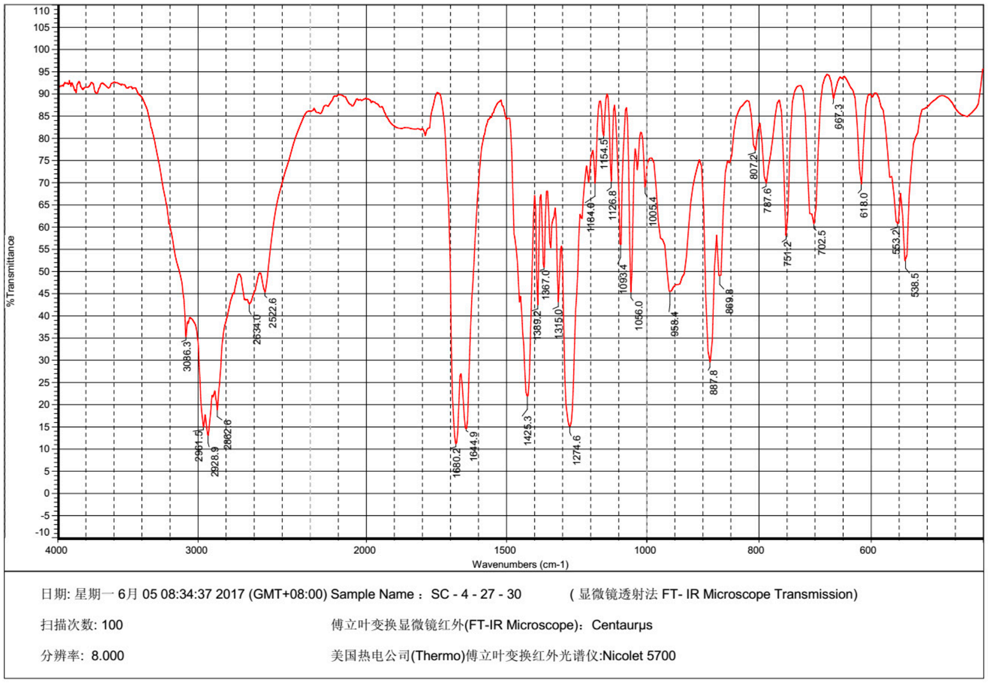

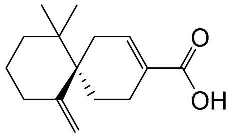

Figure S11. IR spectrum of (6*R*)-*β*-chamigrenic acid (2)

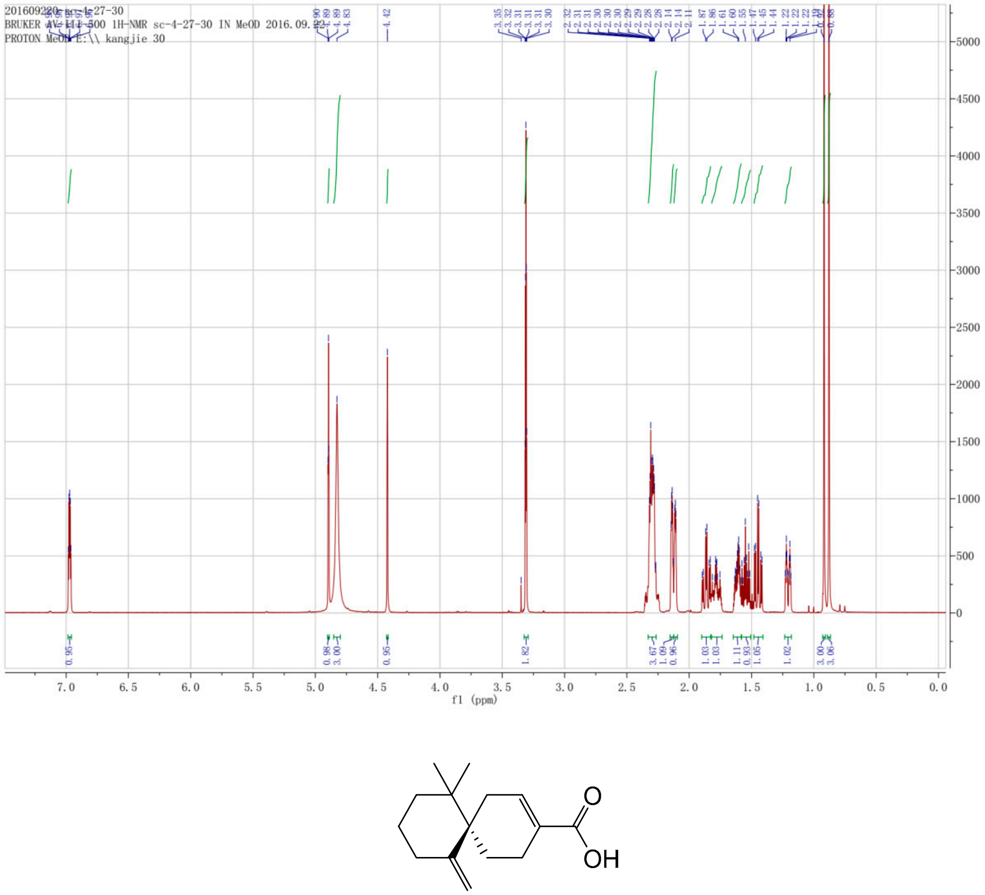

Figure S12. ^{1}H NMR spectrum of (6*R*)-*β*-chamigrenic acid (2; 600 MHz, CD_3OD)

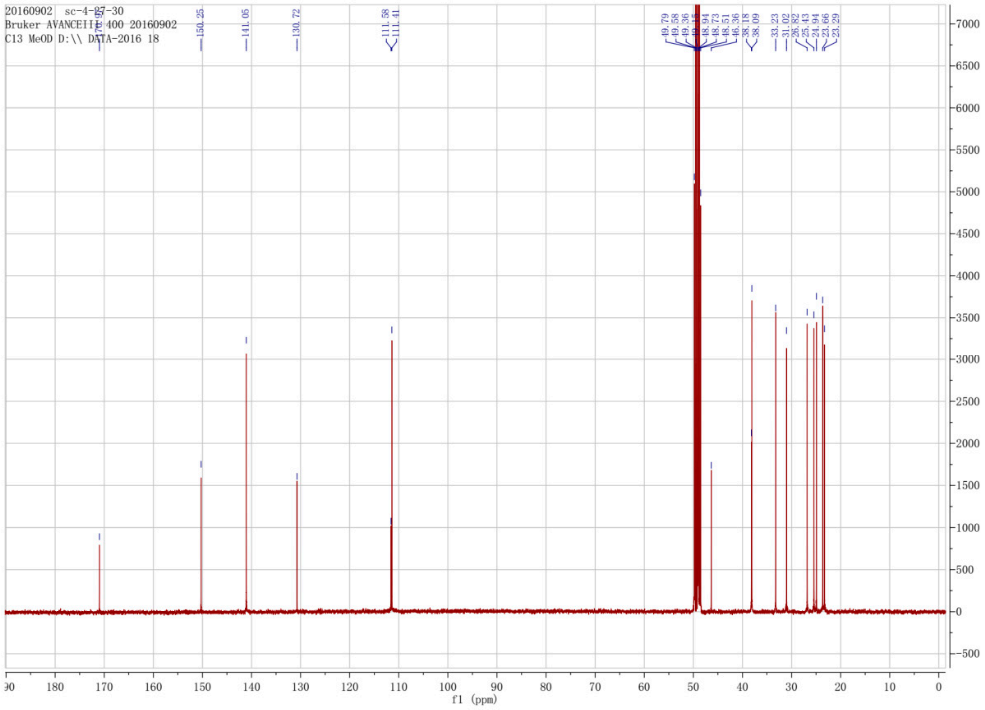

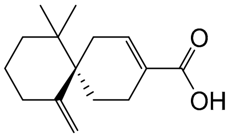

Figure S13. ^{13}C NMR spectrum of (6*R*)-*β*-chamigrenic acid (2; 150 MHz, CD_3OD)

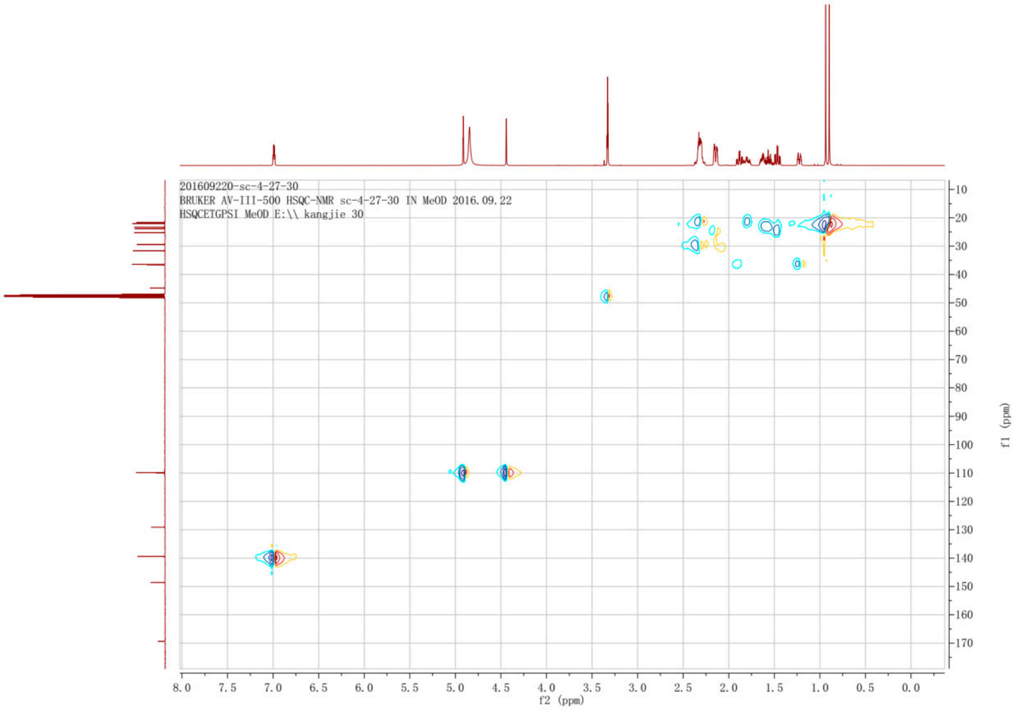

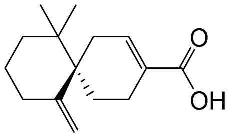

Figure S14. HSQC spectrum of (6*R*)-*β*-chamigrenic acid (2; CD_3OD)

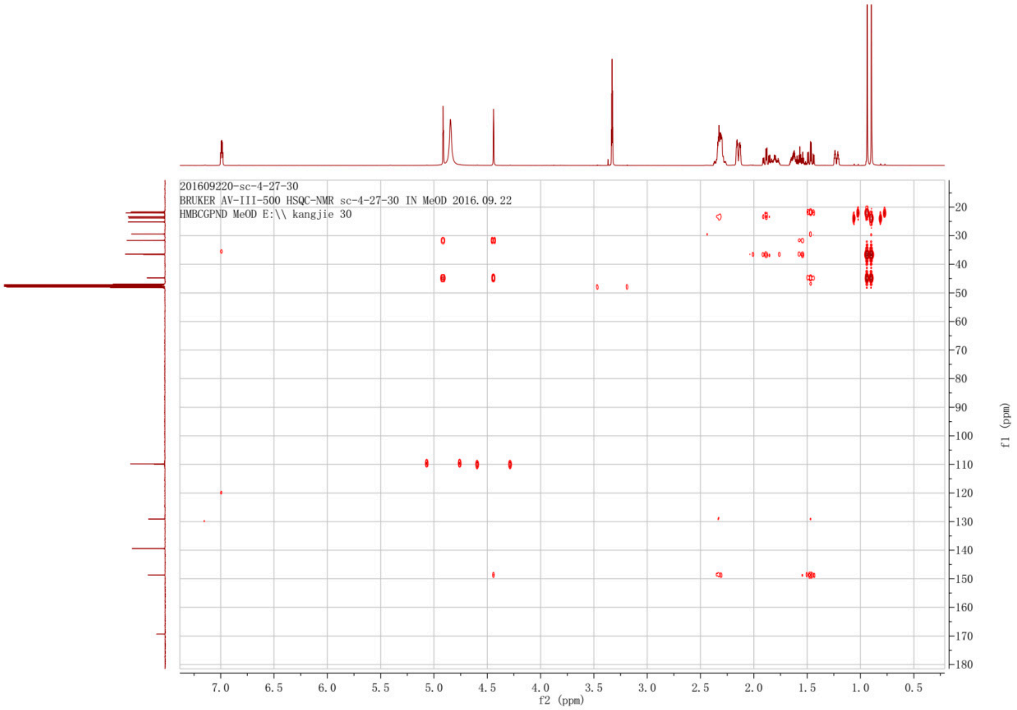

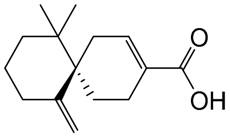

Figure S15. HMBC spectrum of (6*R*)-*β*-chamigrenic acid (2; CD_3OD)
